# Donor-Site Morbidity Following Radial Forearm Free Flap Harvest for Head and Neck Reconstruction: A Systematic Review and Meta-Analysis

**DOI:** 10.3390/jcm15176513

**Published:** 2026-08-23

**Authors:** Fabio Maglitto, Giovanni Salzano, Eutilia Manzo, Serena Trotta, Luigi Angelo Vaira, Marzia Petrocelli, Stefania Troise, Giovanni Dell’Aversana Orabona

**Affiliations:** 1 Maxillofacial Surgery Unit, Department of Neurosciences, Reproductive and Odontostomatological Sciences, University of Naples Federico II, 80131 Naples, Italy; fabio.maglitto@aoufedericoii.unina.it (F.M.); giovannisalzanomd@gmail.com (G.S.); serenatrotta99@icloud.com (S.T.); stefania.troise@unina.it (S.T.); giovanni.dellaversanaorabona@unina.it (G.D.O.); 2 Maxillofacial Surgery Unit, University Hospital of Sassari, 07100 Sassari, Italy; lavaira@uniss.it; 3 Oral and Maxillofacial Unit, USL Bologna Bellaria–Hospital, 40139 Bologna, Italy; marzia.petrocelli@ausl.bologna.it

**Keywords:** radial forearm free flap, donor-site morbidity, head and neck reconstruction, systematic review, meta-analysis

## Abstract

**Background:** The radial forearm free flap (RFFF) remains one of the most reliable and widely adopted reconstructive options for head and neck defects because of its consistent vascular anatomy, long pedicle, and excellent tissue pliability. Nevertheless, donor-site morbidity continues to represent its principal drawback and may negatively affect postoperative recovery, upper-limb function, and patient satisfaction. Although numerous surgical modifications and donor-site reconstruction techniques have been proposed to reduce morbidity, the available evidence remains fragmented and heterogeneous. This systematic review and meta-analysis aimed to quantify the incidence of the principal donor-site complications following RFFF harvest and critically evaluate the current evidence regarding strategies to minimize donor-site morbidity. **Methods:** This systematic review and meta-analysis was conducted according to the PRISMA 2020 statement and prospectively registered in PROSPERO (CRD420261423543). PubMed/MEDLINE, Embase, and Scopus were systematically searched from inception to June 2026. Clinical studies reporting donor-site outcomes after RFFF harvest for head and neck reconstruction were eligible. Primary outcomes were tendon exposure and skin graft loss/failure. Random-effects meta-analyses were performed to estimate pooled incidence rates. Secondary complications, functional outcomes, and patient-reported outcome measures were synthesized qualitatively. Methodological quality and certainty of evidence were assessed using the Joanna Briggs Institute critical appraisal tools and the GRADE framework. **Results:** Twenty-two studies met the inclusion criteria for qualitative synthesis. Fourteen studies were included in the meta-analysis of tendon exposure and twelve in the meta-analysis of graft loss/failure. The pooled incidence of tendon exposure was 8% (95% CI, 7–11%; I^2^ = 0%), whereas the pooled incidence of graft loss/failure was 11% (95% CI, 7–16%; I^2^ = 67.1%). Delayed wound healing, infection, sensory disturbances, scar-related morbidity, and functional impairment were reported inconsistently across studies, precluding quantitative synthesis. Available studies generally reported limited long-term functional impairment, although substantial heterogeneity in assessment methods and follow-up precluded quantitative synthesis. Overall certainty of evidence was rated as low. **Conclusions:** Donor-site morbidity following RFFF harvest remains clinically relevant despite the excellent reconstructive reliability of the flap. Approximately one in twelve patients experiences tendon exposure and one in ten experiences graft loss or failure. Current evidence does not support the superiority of any specific donor-site reconstruction technique. Future high-quality prospective comparative studies adopting standardized outcome definitions and validated patient-reported measures are required to optimize donor-site management and improve reconstructive decision-making.

## 1. Introduction

The radial forearm free flap (RFFF) remains one of the most widely used reconstructive options for head and neck defects following ablative oncologic surgery. Owing to its constant vascular anatomy, long vascular pedicle, thin and pliable soft tissue, and consistently high reliability, the RFFF has become a cornerstone of microsurgical reconstruction for defects involving the oral cavity, tongue, floor of the mouth, oropharynx, and other complex head and neck subsites. Its versatility allows reliable restoration of speech, swallowing, and oral competence while maintaining excellent reconstructive success rates. Despite these well-established advantages, donor-site morbidity continues to represent the principal limitation of the RFFF and remains a major determinant of postoperative recovery, upper-limb function, cosmetic outcome, and patient satisfaction [1].

Harvest of the radial forearm flap inevitably creates a forearm defect that usually requires skin grafting or alternative reconstructive techniques. Consequently, patients may experience a broad spectrum of donor-site complications, including tendon exposure, partial or complete graft failure, delayed wound healing, infection, sensory disturbances, reduced grip strength, restricted wrist mobility, hypertrophic scarring, neuropathic pain, and aesthetic dissatisfaction [1,2]. Although these complications rarely compromise flap survival, they may substantially affect upper-extremity function and health-related quality of life. Therefore, optimization of donor-site management should be regarded as an essential component of reconstructive success rather than merely a secondary technical consideration.

Over the past two decades, numerous surgical strategies have been proposed to minimize donor-site morbidity [2,3,4]. Technical refinements during flap harvest, particularly suprafascial elevation with preservation of the paratenon, have been associated with improved graft take and lower rates of tendon exposure [5,6]. Likewise, several donor-site closure techniques—including split-thickness skin grafts (STSGs), full-thickness skin grafts (FTSGs), dermal regeneration templates, acellular dermal matrices, negative-pressure wound therapy, local and regional flaps, and primary closure techniques—have been introduced to improve wound healing while reducing functional impairment and aesthetic sequelae [3,7,8,9,10,11,12].

Among these strategies, the optimal method for donor-site closure remains controversial. In recent years, three independent systematic reviews and meta-analyses have specifically compared STSGs and FTSGs, consistently concluding that the available evidence is limited by the predominance of retrospective studies, heterogeneous surgical techniques, inconsistent outcome definitions, and relatively small sample sizes [13,14,15]. Although some authors have reported potential advantages of FTSGs in selected aesthetic outcomes, whereas others observed lower rates of graft-related complications with STSGs, no high-quality evidence currently supports the routine use of one grafting technique over the other [13,14,15]. Similarly, contemporary cohort studies investigating modified closure techniques, dermal substitutes, and innovative reconstructive approaches have shown encouraging results but remain limited by methodological heterogeneity and the absence of standardized outcome reporting [8,10,11,12,16,17,18].

Despite the growing body of literature, several important knowledge gaps remain. Most published studies are retrospective, involve relatively small patient cohorts, and evaluate heterogeneous donor-site reconstruction techniques using variable follow-up protocols and non-standardized definitions of donor-site complications. Furthermore, clinically relevant outcomes—including tendon exposure, graft loss or failure, sensory dysfunction, patient-reported functional outcomes, and aesthetic satisfaction—are frequently reported as secondary endpoints, making meaningful comparisons across studies difficult [2,3,4,12,13,14,15,16,17]. Consequently, clinicians still lack robust pooled estimates of the principal donor-site complications and high-quality comparative evidence capable of guiding evidence-based donor-site reconstruction.

Therefore, the aim of this systematic review and meta-analysis was to quantify the incidence of the principal donor-site complications following radial forearm free flap harvest for head and neck reconstruction and to critically evaluate the current evidence regarding surgical strategies designed to minimize donor-site morbidity. In addition, secondary donor-site complications, functional outcomes, patient-reported outcome measures, and the certainty of the available evidence were systematically assessed to provide a comprehensive overview of contemporary donor-site management.

## 2. Materials and Methods

### 2.1. Study Protocol

This systematic review and meta-analysis was conducted in accordance with the Preferred Reporting Items for Systematic Reviews and Meta-Analyses (PRISMA) 2020 Statement (Supplementary Table S1) [19]. The study protocol was prospectively registered in the International Prospective Register of Systematic Reviews (PROSPERO) under registration number CRD420261423543 (https://www.crd.york.ac.uk/PROSPERO/view/CRD420261423543; accessed on 15 June 2026), thereby ensuring methodological transparency and minimizing the risk of selective reporting. The present review was conducted according to the prospectively registered protocol, with no substantive deviations from the prespecified methodology. The review aimed to evaluate donor-site morbidity following radial forearm free flap (RFFF) harvest in patients undergoing head and neck reconstruction and to critically assess the available evidence regarding surgical strategies intended to reduce donor-site complications. The research question was developed according to the Population, Intervention, Comparison, Outcome (PICO) framework: Population: patients undergoing radial forearm free flap harvest for head and neck reconstruction; Intervention: surgical techniques designed to reduce donor-site morbidity, including modifications of flap harvest, donor-site closure methods, dermal substitutes, negative-pressure wound therapy, and local flap reconstruction; Comparison: conventional donor-site management or alternative reconstructive techniques; and Outcomes: donor-site morbidity, including tendon exposure, graft loss or failure, wound complications, functional impairment, scar quality, aesthetic outcomes, and patient-reported outcome measures. Two reviewers independently performed study selection, data extraction, and methodological quality assessment. Disagreements were resolved through discussion until consensus was reached.

### 2.2. Search Strategy

A comprehensive literature search was performed to identify studies evaluating donor-site morbidity following radial forearm free flap harvest in head and neck reconstruction. The search strategy combined controlled vocabulary terms and free-text keywords related to radial forearm free flaps, head and neck reconstruction, donor-site morbidity, functional outcomes, and wound complications, which were searched within titles and abstracts to maximize sensitivity. The following electronic databases were systematically searched from inception to June 2026: PubMed/MEDLINE, Embase and Scopus. Only studies published in English were considered eligible. The search strategy was adapted to the specific syntax and controlled vocabulary of each database. The complete search strategies for all electronic databases are reported in Supplementary S1.

### 2.3. Study Selection and Data Collection Process

All records identified through the database searches were imported into EndNote™ Web (Clarivate Plc, Philadelphia, PA, USA) (https://access.clarivate.com/login?app=endnote, accessed on 14 June 2026) for management and duplicate removal. Both automated and manual deduplication procedures were performed before screening. Study selection was conducted in two sequential phases. First, titles and abstracts were independently screened by two reviewers according to the predefined eligibility criteria. Studies clearly unrelated to donor-site morbidity following radial forearm free flap harvest were excluded at this stage. In the second phase, the full texts of potentially eligible articles were independently assessed for inclusion. Studies were considered eligible if they: (1) included adult patients undergoing radial forearm free flap harvest for head and neck reconstruction; (2) reported at least one donor-site outcome of interest (e.g., tendon exposure, graft loss/failure, wound complications, functional outcomes, aesthetic outcomes, or patient-reported outcome measures); and (3) were original clinical studies, including randomized controlled trials, prospective or retrospective cohort studies, comparative studies, cross-sectional studies, and case series. Studies including mixed reconstructive populations were eligible only if donor-site outcomes for radial forearm free flaps used in head and neck reconstruction could be extracted separately. No minimum follow-up duration or minimum sample size was prespecified because the review aimed to capture both early postoperative complications and long-term donor-site outcomes. No minimum study sample size was prespecified. Case reports describing individual patients were excluded, whereas case series were eligible irrespective of sample size provided that they fulfilled all other eligibility criteria. When overlapping patient cohorts were identified, only the most comprehensive or most recent study was included. Reviews, case reports, conference abstracts, editorials, letters to the editor, narrative reviews, systematic reviews, animal studies, cadaveric studies, and articles not published in English were excluded. Data extraction was independently performed by two reviewers using a standardized data collection form. Extracted variables included first author, year of publication, country, study design, sample size, patient characteristics, donor-site management technique, comparator group when available, follow-up duration, donor-site complications, functional outcomes, aesthetic outcomes, and patient-reported outcome measures. Detailed study-level information, including donor-site reconstruction techniques, study design, sample size, follow-up, outcome-specific denominators, and reported donor-site complications, is provided in Supplementary Table S3. Any discrepancies during study selection or data extraction were resolved through discussion and consensus.

### 2.4. Outcomes and Definitions

The primary outcomes of this systematic review were donor-site wound complications following radial forearm free flap harvest, with particular focus on two outcomes selected for quantitative synthesis: tendon exposure and skin graft loss or graft failure. Tendon exposure was defined as any postoperative exposure of the underlying tendons at the forearm donor site, regardless of whether additional surgical management was required. Skin graft loss/failure was defined as any explicitly reported partial or complete loss, necrosis, or incomplete take of a skin graft used for donor-site coverage. The denominator for this outcome was restricted, whenever extractable, to donor sites that received skin graft reconstruction. Because the included studies used heterogeneous terminology and generally did not apply standardized severity thresholds or assessment time points, events were classified according to the definitions provided by the original investigators. Outcomes reported only as nonspecific wound breakdown or delayed wound healing, without sufficient information to establish graft involvement, were not included in the quantitative synthesis. Partial and complete graft failure were distinguished whenever this information was explicitly reported by the original studies; however, separate quantitative pooling according to severity was not feasible because severity-specific data were inconsistently reported. Additional donor-site outcomes included delayed wound healing, wound dehiscence, donor-site infection, hematoma or seroma, hypertrophic scarring, sensory disturbances, reduced grip strength, impaired wrist range of motion, scar quality, aesthetic outcomes, patient satisfaction, and patient-reported functional outcomes, including Disabilities of the Arm, Shoulder and Hand scores when available. For studies reporting multiple donor-site outcomes, all relevant variables were extracted. Outcomes not suitable for quantitative pooling because of inconsistent definitions, heterogeneous reporting, or insufficient extractable data were synthesized narratively. For secondary outcomes, the definitions adopted by the original studies were retained because standardized definitions could not be applied retrospectively. For example, delayed wound healing variably encompassed epidermolysis, wound breakdown, graft separation, or prolonged time to complete epithelialization, whereas scar-related outcomes included hypertrophic scarring, contracture, unstable scars, hypergranulation, and subjective cosmetic dissatisfaction assessed using different methods. Functional outcomes were likewise evaluated using heterogeneous measures, including DASH scores, grip strength, wrist range of motion, sensory testing, and patient-reported symptoms at variable follow-up intervals. This clinical and methodological heterogeneity precluded meaningful quantitative pooling of these secondary outcomes.

### 2.5. Risk of Bias Assessment

**Figure 1:**
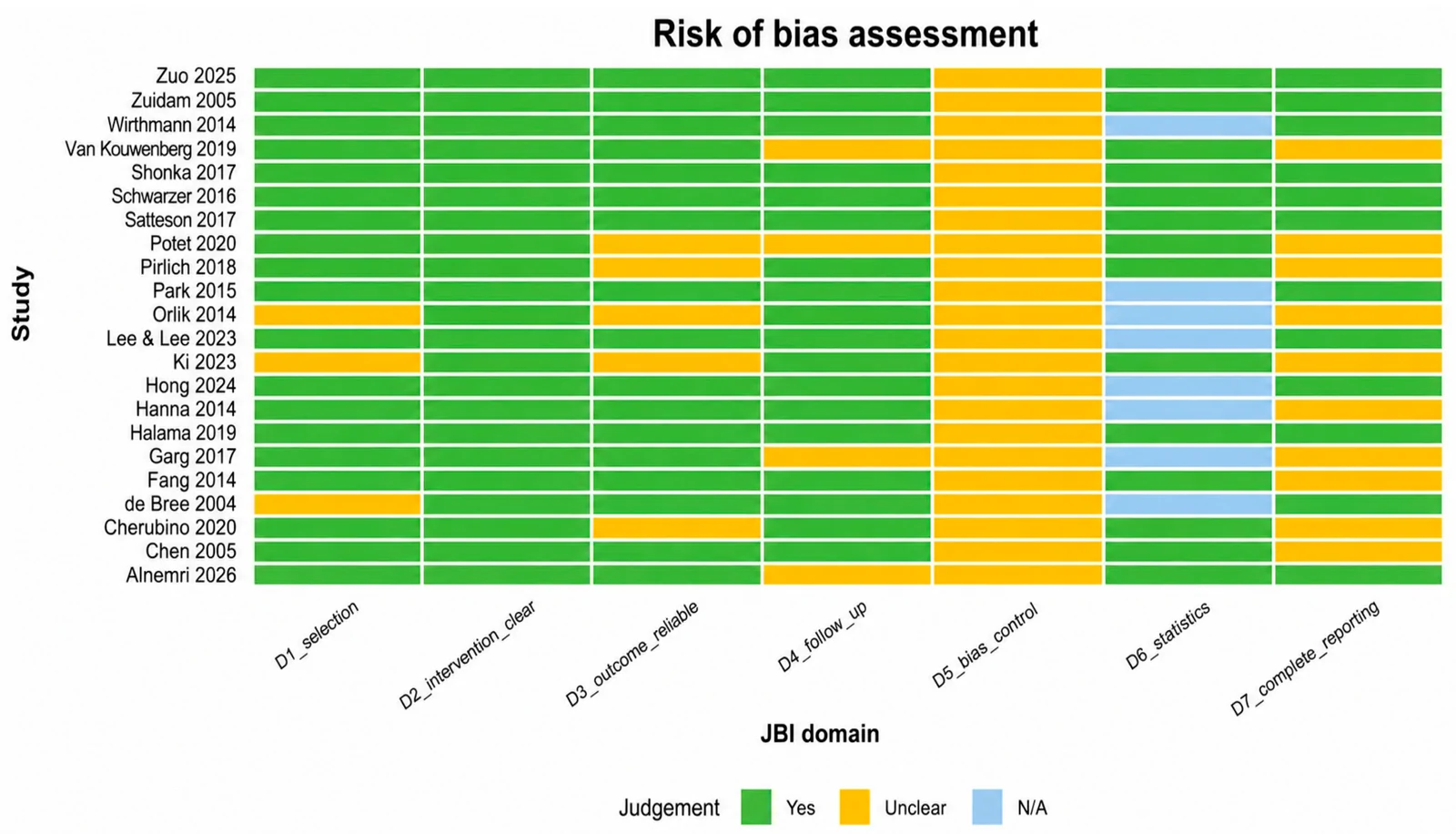
Risk of bias assessment of the included studies according to the Joanna Briggs Institute (JBI) critical appraisal tools [20]. Traffic-light plot showing the methodological quality of each included study across seven harmonized domains: selection/inclusion criteria (D1), description of intervention (D2), reliability of outcome assessment (D3), adequacy of follow-up (D4), control of confounding or consecutive inclusion when applicable (D5), appropriateness of statistical analysis (D6), and completeness of donor-site outcome reporting (D7). Green indicates low risk of bias (Yes), yellow indicates unclear risk of bias (Unclear), and blue indicates domains not applicable (N/A) The assessment includes the studies by Ki et al. (2023) [5], Shonka et al. (2017) [6], Alnemri et al. (2026) [18], Schwarzer et al. (2016) [21], Garg et al. (2017) [22], Cherubino et al. (2020) [7], Potet et al. (2020) [8], Zuidam et al. (2005) [23], Van Kouwenberg et al. (2019) [24], Satteson et al. (2017) [25], Park et al (2015) [9], Hong (2024) [26], Chen et al. (2005) [27], Wirthmann et al. (2014) [28], Orlik et al. (2014) [29], de Bree et al. (2004) [30], Lee & Lee (2023) [31], Hanna et al. (2014) [32], Zuo et al. (2025) [12], Halama et al. (2019) [10], Pirlich et al. (2018) [33], and Fang et al. (2014) [34].

The methodological quality of the included studies was assessed using Joanna Briggs Institute (JBI) critical appraisal tools according to study design [20]. Randomized controlled trials were evaluated using the JBI checklist for randomized controlled trials, whereas observational comparative studies, cohort studies, cross-sectional studies, and case series were assessed using the corresponding JBI appraisal tools. For graphical presentation, judgments were harmonized across seven domains: selection/inclusion criteria, description of participants and intervention, reliability of outcome assessment, adequacy of follow-up, control of confounding or consecutive inclusion when applicable, appropriateness of statistical analysis, and completeness of donor-site outcome reporting. Each domain was rated as “Yes,” “No,” “Unclear,” or “Not applicable,” and the results were summarized graphically using harmonized JBI domains across study designs (Figure 1 and Supplementary Figure S7). Disagreements were resolved by consensus.

### 2.6. Statistical Analysis

All statistical analyses were performed using R software (version 4.6.0; R Foundation for Statistical Computing, Vienna, Austria) and the meta package (version 8.5-0) for R. A two-sided *p* value < 0.05 was considered statistically significant. A qualitative synthesis was performed for all included studies. Quantitative synthesis was undertaken when at least two studies reported extractable data for the same donor-site outcome using sufficiently comparable outcome definitions. Two separate meta-analyses were performed to estimate the pooled incidence of tendon exposure and skin graft loss/failure following RFFF donor-site reconstruction. The donor site represented the unit of analysis. For graft loss/failure, denominators were restricted to grafted donor sites whenever these data could be separately identified. Comparative pooling according to donor-site reconstruction technique was not performed because the available studies differed substantially in reconstruction strategy, comparator definition, patient characteristics, follow-up duration, and outcome ascertainment, and the number of studies evaluating individual techniques was insufficient for clinically meaningful subgroup analyses or meta-regression. Combining these heterogeneous comparative groups could therefore have generated misleading estimates of relative treatment effects. Accordingly, pooled incidence estimates across eligible studies were considered the most methodologically appropriate quantitative synthesis, while evidence concerning differences among reconstruction techniques was synthesized narratively. For each eligible study, the number of events and the corresponding number of donor sites at risk were extracted. Pooled proportions and corresponding 95% confidence intervals (95% CIs) were estimated using a random-intercept logistic regression model with a logit transformation, implemented using the metaprop function of the meta package. This approach was selected because it allows the synthesis of proportions while appropriately accommodating studies with low or zero event counts. Between-study heterogeneity was assessed using Cochran’s Q statistic, with its corresponding degrees of freedom and *p* value, the I^2^ statistic with 95% CI, and the between-study variance (τ^2^), estimated using the maximum-likelihood (ML) method on the logit scale. For descriptive interpretation, I^2^ values of approximately 25%, 50%, and 75% were considered indicative of low, moderate, and high statistical heterogeneity, respectively; however, these thresholds were not interpreted rigidly and were considered together with the 95% CI for I^2^, the magnitude and direction of study estimates, and the clinical and methodological heterogeneity of the included studies. A non-significant Cochran Q test or an I^2^ estimate of 0% was not considered evidence of absence of heterogeneity, particularly when the number of included studies was limited. Prediction intervals were calculated using a t-distribution to estimate the expected range of true complication rates in future comparable clinical settings. Potential small-study effects were evaluated by visual inspection of funnel plots and Egger’s regression test for outcomes including at least ten studies. Funnel-plot asymmetry and statistically significant Egger tests were interpreted as evidence of possible small-study effects rather than publication bias alone, because asymmetry in meta-analyses of proportions may also arise from differences in study size, event frequency, clinical or methodological heterogeneity, and selective outcome reporting. Sensitivity analyses were performed using leave-one-out influence analyses to evaluate the robustness of pooled estimates after sequential exclusion of each individual study. Subgroup analyses and meta-regression were not performed because of the limited number of studies within individual donor-site management strategies and the substantial clinical heterogeneity of the available evidence.

### 2.7. Certainty of Evidence Assessment

The certainty of evidence for the primary outcomes was evaluated using the Grading of Recommendations Assessment, Development and Evaluation (GRADE) framework. Evidence was assessed across the domains of risk of bias, inconsistency, indirectness, imprecision, and publication bias. A Summary of Findings table was generated for both primary outcomes (Supplementary Table S2).

## 3. Results

### 3.1. Study Selection

**Figure 2:**
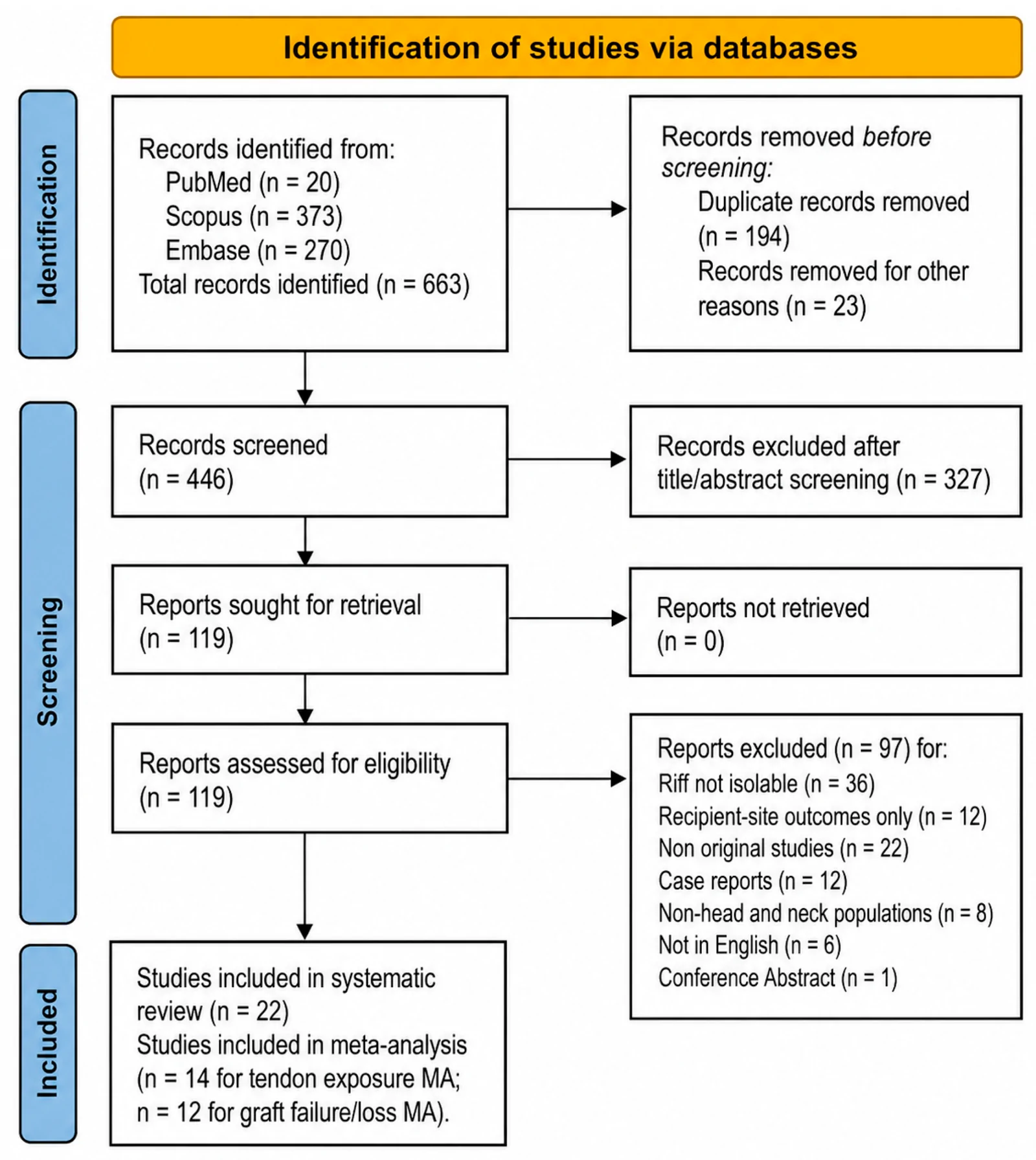
PRISMA flow diagram illustrating the study selection process according to PRISMA 2020 guidelines, including identification, screening, eligibility, and inclusion of studies for the qualitative and quantitative synthesis [19].

The literature search identified 663 records from three electronic databases (PubMed, *n* = 20; Scopus, *n* = 373; Embase, *n* = 270). After removal of duplicate records (*n* = 194) and records removed for other reasons (*n* = 23), 446 records remained for title and abstract screening. Of these, 327 records were excluded following title and abstract screening, and 119 reports were assessed for eligibility. Of these, 97 reports were excluded for the following reasons: inability to isolate radial forearm free flap outcomes from mixed reconstructive cohorts (*n* = 36), non-original studies (*n* = 22), recipient-site outcomes only (*n* = 12), case reports (*n* = 12), non-head and neck populations (*n* = 8), articles not published in English (*n* = 6), and conference abstracts (*n* = 1). Ultimately, 22 studies met the inclusion criteria and were included in the qualitative synthesis. Fourteen studies provided extractable data for the meta-analysis of tendon exposure, whereas twelve studies were eligible for the meta-analysis of graft loss/failure. The study selection process is summarized in Figure 2.

### 3.2. Study Characteristics

The 22 included studies comprised predominantly retrospective observational investigations evaluating donor-site outcomes following RFFF harvest for head and neck reconstruction. Sample sizes ranged from 5 to 179 donor sites, and follow-up duration varied substantially across studies, ranging from the early postoperative period to long-term follow-up exceeding eight years. A wide variety of donor-site management strategies were investigated, including split-thickness and full-thickness skin grafts, dermal substitutes, negative-pressure wound therapy, primary closure techniques, and local flap reconstruction. Although tendon exposure and graft loss/failure were the most consistently reported complications, considerable variability existed in the assessment of secondary donor-site complications, functional recovery, scar quality, and patient-reported outcomes. The principal characteristics of the included studies are summarized in Table 1, while detailed study-level information, including donor-site reconstruction strategies, follow-up, outcome-specific denominators, and additional donor-site complications, is provided in Supplementary Table S3.

### 3.3. Risk of Bias Results

The overall methodological quality of the included studies was moderate, although several limitations related to the predominance of retrospective observational designs were identified (Figure 2; Supplementary Figure S7). Selection criteria were adequately described in most studies, with 91% fulfilling the corresponding JBI domain, while the remaining 9% provided insufficient information regarding patient selection. All studies clearly described the surgical intervention and perioperative management (100%), and outcome assessment was judged to be appropriate in 82% of studies, with the remaining 18% judged as unclear due to insufficient reporting of outcome definitions or assessment methods. Adequacy of follow-up was satisfactory in 68% of studies but remained unclear in 32%, primarily because follow-up duration or completeness was insufficiently reported. The domain related to control of confounding factors or consecutive patient inclusion represented the principal methodological limitation, reflecting the predominance of retrospective non-randomized study designs, with only 14% of studies fulfilling this criterion and 82% judged as unclear because of insufficient control of potential confounding factors. This domain was considered not applicable in the remaining studies according to the corresponding JBI checklist. Statistical analyses were appropriate in 82% of studies, whereas this domain was not applicable in 18%, mainly in descriptive case series. Completeness of donor-site outcome reporting was adequate in 68% of studies but unclear in 32%, reflecting incomplete reporting of secondary donor-site complications or functional outcomes. Overall, the included studies demonstrated moderate methodological quality, with the greatest methodological uncertainty relating to incomplete follow-up reporting, limited control of confounding variables, and inconsistent reporting of secondary donor-site outcomes.

### 3.4. Meta-Analysis of Tendon Exposure

**Figure 3:**
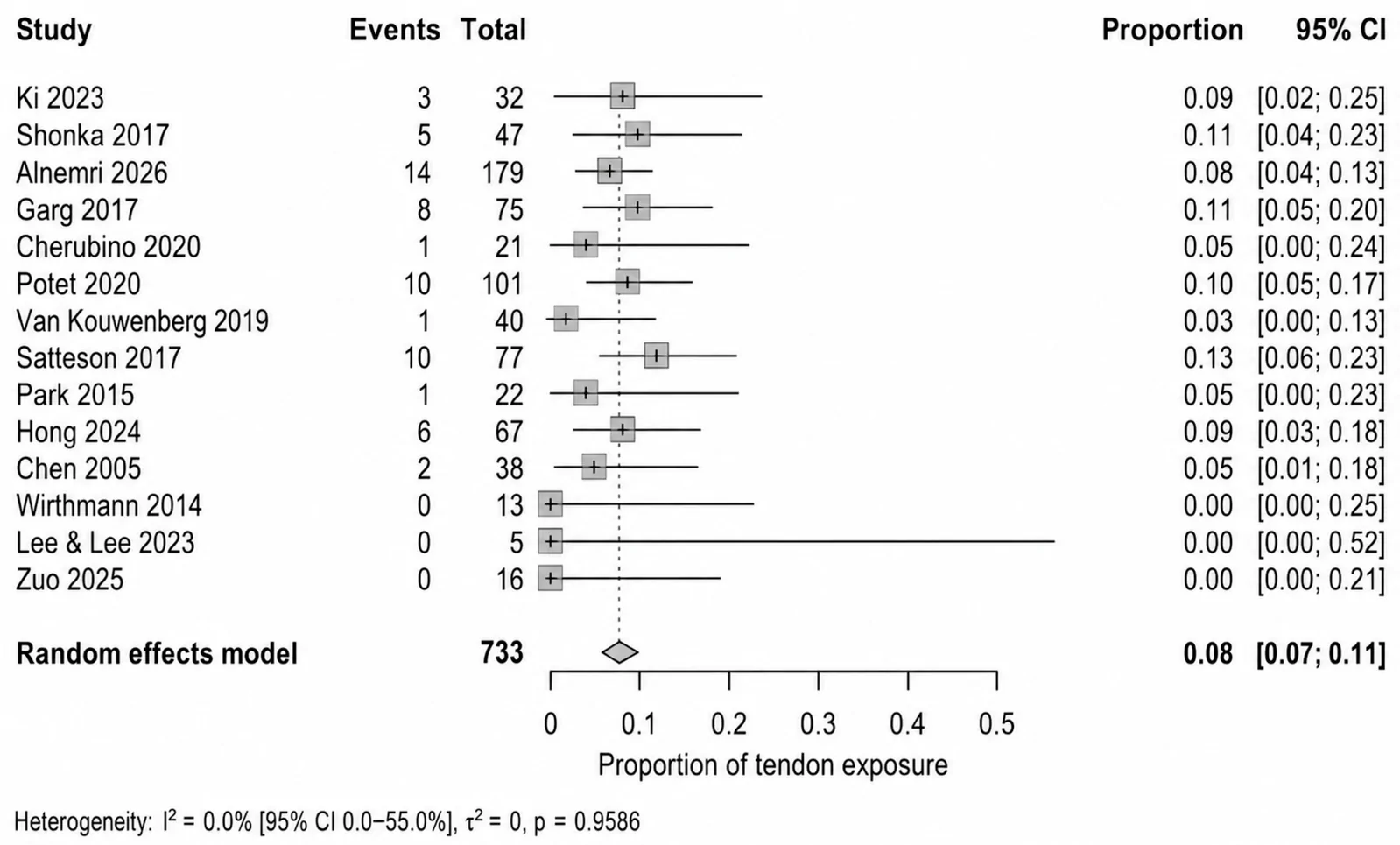
Forest plot showing the pooled incidence of tendon exposure after radial forearm free flap donor-site reconstruction across 14 studies comprising 733 donor sites. Squares represent individual study estimates, horizontal lines indicate 95% confidence intervals, and the diamond represents the pooled proportion estimated using a random-effects model. The pooled incidence was 8.32% (95% CI 6.53–10.55%), with I^2^ = 0.0% (95% CI 0.0–55.0%). The meta-analysis includes the studies by Ki et al. (2023) [5], Shonka et al. (2017) [6], Alnemri et al. (2026) [18], Garg et al. (2017) [22], Cherubino et al. (2020) [7], Potet et al. (2020) [8], Van Kouwenberg et al. (2019) [24], Satteson et al. (2017) [25], Park et al. (2015) [9], Hong (2024) [26], Chen et al. (2005) [27], Wirthmann et al. (2014) [28], Lee & Lee (2023) [31], and Zuo et al. (2025) [12].

Fourteen studies comprising 733 radial forearm free flap donor sites were eligible for quantitative synthesis of tendon exposure. Across all included studies, 61 tendon exposure events were reported. The pooled analysis using a random-effects model demonstrated an overall tendon exposure incidence of 8% (95% CI, 7–11%) (Figure 3). Individual study estimates ranged from 0% to 13%, while most studies reported tendon exposure rates below 10%. The point estimate of statistical heterogeneity was low (I^2^ = 0.0%, 95% CI 0.0–55.0%; τ^2^ = 0), although the confidence interval around I^2^ indicates that clinically relevant between-study heterogeneity cannot be excluded. Cochran’s Q test was not statistically significant (Q = 5.63, df = 13, *p* = 0.9586). To estimate the expected range of results in future comparable studies, a prediction interval was calculated. The 95% prediction interval ranged from 6.37% to 10.81%, indicating that the expected incidence in a future comparable clinical setting would remain relatively close to the pooled estimate, while acknowledging the uncertainty associated with between-study heterogeneity (Supplementary Figure S1). The robustness of the pooled estimate was further evaluated through leave-one-out sensitivity analysis. Sequential exclusion of each individual study produced pooled incidences ranging from 8% to 9%, with no meaningful change in the overall estimate or heterogeneity (I^2^ remained 0% throughout all iterations), indicating that no single study exerted a disproportionate influence on the pooled result (Supplementary Figure S2). Potential small-study effects were explored visually using a funnel plot and statistically using Egger’s regression test. Visual inspection suggested funnel-plot asymmetry (Supplementary Figure S3), and Egger’s regression test indicated statistically significant small-study effects (t = −3.10, *p* = 0.009). However, this finding should not be interpreted as evidence of publication bias alone, as funnel-plot asymmetry in single-arm proportion meta-analyses may also reflect differences in study size, event frequency, methodological characteristics, or selective outcome reporting.

### 3.5. Meta-Analysis of Graft Loss/Failure

**Figure 4:**
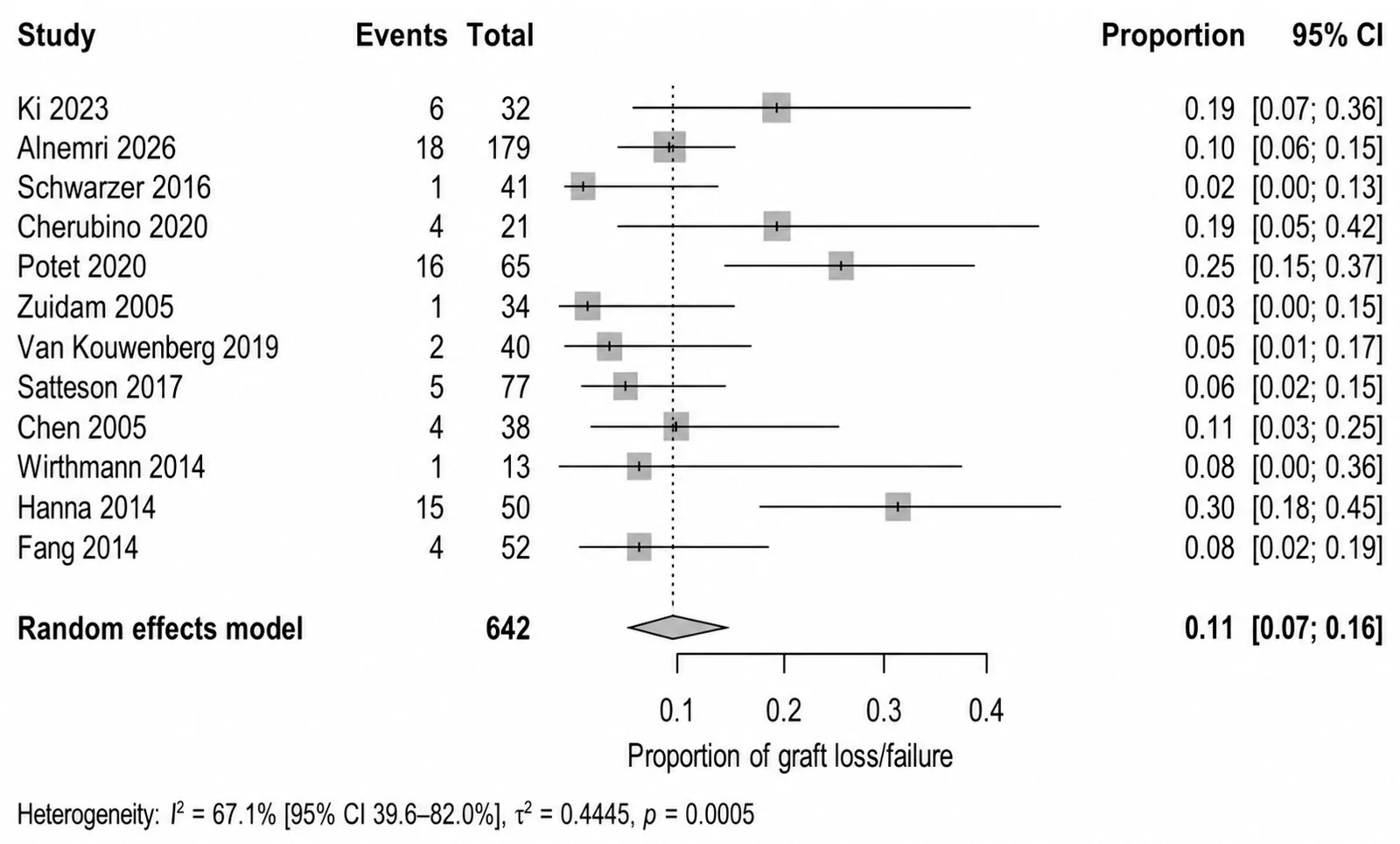
Forest plot showing the pooled incidence of graft loss/failure following radial forearm free flap donor-site reconstruction across 12 studies comprising 642 donor sites. Squares represent individual study estimates, horizontal lines indicate 95% confidence intervals, and the diamond represents the pooled proportion estimated using a random-effects model. The pooled incidence was 10.56% (95% CI 6.73–16.19%), with substantial heterogeneity (I^2^ = 67.1%, 95% CI 39.6–82.0%). The meta-analysis includes the studies by Ki et al. (2023) [5], Cherubino et al. (2020) [7], Potet et al. (2020) [8], Alnemri et al. (2026) [18], Schwarzer et al. (2016) [21], Zuidam et al. (2005) [23], Van Kouwenberg et al. (2019) [24], Satteson et al. (2017) [25], Chen et al. (2005) [27], Wirthmann et al. (2014) [28], Hanna et al. (2014) [32], and Fang et al. (2014) [34].

Data on graft loss/failure were available from 12 studies, including a total of 642 donor sites and 77 graft failures. The pooled incidence of graft loss/failure was 11% (95% CI, 7–16%) using a random-effects model (Figure 4). Substantial between-study heterogeneity was observed (I^2^ = 67.1%, 95% CI 39.6–82.0%; τ^2^ = 0.4445 on the logit scale), with a statistically significant Cochran’s Q test (Q = 33.40, df = 11, *p* = 0.0005). The 95% prediction interval ranged from 2.40% to 36.15% (Supplementary Figure S4), indicating considerable variability in the underlying incidence across comparable clinical settings. Sensitivity analysis using the leave-one-out approach demonstrated that the pooled estimate remained stable after sequential exclusion of each individual study (Supplementary Figure S5). The pooled incidence ranged from 9% to 12%, indicating that no single study exerted a disproportionate influence on the overall estimate. Although exclusion of the study by Hanna et al. modestly reduced heterogeneity (I^2^ from 67.1% to 54.9%), the overall findings remained unchanged. Visual inspection of the funnel plot did not reveal marked asymmetry (Supplementary Figure S6), and Egger’s regression test was not statistically significant (t = −2.03, *p* = 0.070). However, given the limited number of studies and the characteristics of proportion meta-analysis, the absence of statistical significance should not be interpreted as definitive evidence against small-study effects or selective reporting.

### 3.6. Secondary Donor-Site Complications

Secondary donor-site complications were reported heterogeneously across the included studies and were therefore not suitable for quantitative synthesis. The most frequently reported complications included delayed wound healing, infection, hematoma, seroma, scar-related morbidity, sensory disturbances, and the need for secondary interventions. Delayed wound healing was among the most commonly reported complications, although its incidence varied considerably across studies, whereas some studies evaluating primary closure techniques reported complete donor-site healing without delayed wound complications. Donor-site infection was generally uncommon, while hematoma and seroma were reported infrequently. Scar-related morbidity encompassed hypertrophic scarring, scar contracture, unstable scars, hypergranulation tissue, epidermolysis, and poor cosmetic appearance. However, scar assessment was inconsistently performed, and no standardized scar evaluation tool was uniformly adopted across studies, limiting direct comparison of outcomes. Sensory disturbances were among the most frequently reported long-term sequelae and included numbness, paresthesia, dysesthesia, reduced sensibility, cold intolerance, and abnormal sensation, with occasional reports of complex regional pain syndrome. The need for secondary donor-site procedures was uncommon and consisted primarily of secondary skin grafting, debridement, or revision surgery. Overall, the interpretation of secondary donor-site outcomes is limited by substantial heterogeneity in outcome definitions, assessment methods, and reporting across the available studies.

### 3.7. Functional and Patient-Reported Outcomes

Functional recovery and patient-reported outcomes were evaluated using heterogeneous assessment methods, precluding quantitative synthesis. The Disabilities of the Arm, Shoulder and Hand (DASH) questionnaire represented the most frequently adopted validated instrument. Overall, the available studies generally reported limited long-term upper-limb functional impairment following RFFF harvest. However, reductions in grip strength, wrist mobility, and manual dexterity were reported in some cohorts, and the heterogeneity of assessment methods and follow-up intervals limited direct comparison across studies. Assessment of cosmetic outcomes was highly variable. While some studies described residual aesthetic dissatisfaction or scar-related concerns, most reported acceptable long-term donor-site appearance and favorable patient-reported quality-of-life outcomes.

### 3.8. Certainty of Evidence Results

According to the GRADE framework, the certainty of evidence for both primary outcomes was rated as low. For tendon exposure, certainty was downgraded because of the predominance of observational studies and possible small-study effects. For graft loss/failure, evidence was additionally downgraded because of substantial between-study heterogeneity. No factors supporting upgrading of the certainty of evidence were identified. The complete GRADE assessment is reported in Supplementary Table S2.

## 4. Discussion

The present systematic review and meta-analysis provides a comprehensive quantitative evaluation of donor-site morbidity following radial forearm free flap (RFFF) harvest in head and neck reconstruction. By synthesizing the currently available evidence, this study establishes benchmark estimates for the two most consistently reported donor-site complications—tendon exposure and graft loss or failure—while critically appraising the evidence supporting contemporary donor-site management strategies. Although the reconstructive reliability of the RFFF is well established, donor-site morbidity remains one of its principal limitations and continues to influence postoperative recovery, upper-extremity function, cosmetic outcomes, and patient satisfaction. Previous meta-analyses comparing radial and ulnar forearm free flaps have similarly reported no conclusive evidence supporting the superiority of either donor site with respect to donor-site morbidity [35]. Even though alternative flaps, particularly the anterolateral thigh flap, may reduce donor-site morbidity in selected patients, the RFFF remains an indispensable reconstructive option when thin, pliable tissue, a long vascular pedicle, and reliable anatomy are required [36].

Over the past two decades, numerous modifications of flap harvest and donor-site reconstruction have been proposed to minimize donor-site morbidity [3,10]. More recently, three independent systematic reviews and meta-analyses have specifically compared split-thickness skin grafts (STSGs) and full-thickness skin grafts (FTSGs) for donor-site closure [13,14,15]. Despite minor differences in their conclusions, these reviews consistently emphasize the predominance of retrospective study designs, heterogeneous reconstructive techniques, inconsistent outcome definitions, and limited methodological quality, precluding robust recommendations in favor of either grafting strategy. While Saleki et al. suggested that FTSGs may provide modest aesthetic advantages without significantly affecting wound-related complications [15], Zhang et al. reported a higher incidence of graft-related complications associated with FTSGs without demonstrating significant cosmetic benefits [13]. More recently, Moors et al. concluded that the currently available evidence does not support the superiority of either STSGs or FTSGs with respect to wound-related, functional, or aesthetic outcomes and highlighted the need for adequately powered prospective comparative studies [14]. Our findings reinforce these conclusions by demonstrating that meaningful quantitative comparison between donor-site reconstruction strategies remains precluded by substantial methodological heterogeneity and inconsistent outcome reporting.

One of the principal findings of the present meta-analysis is the relatively consistent incidence of tendon exposure across the included studies. Across fourteen studies including more than 700 donor sites, the pooled incidence was approximately 8%, with an I^2^ of 0%. Although this finding provides a robust benchmark estimate, the absence of statistical heterogeneity should not be interpreted as evidence of true clinical homogeneity. Important differences remained across studies regarding patient selection, reconstructive indication, flap harvest technique, donor-site closure method, postoperative wound management, and duration of follow-up. Consequently, the pooled estimate should be regarded as a reference benchmark for the available literature rather than a precise prediction of complication risk applicable to individual patients or institutions.

Several technical principles have been advocated to minimize tendon exposure, including preservation of the paratenon, meticulous soft-tissue handling, maintenance of an adequately vascularized wound bed, and atraumatic graft fixation. However, the present analysis was not designed to determine the independent contribution of these variables, and no causal inference regarding specific surgical techniques can therefore be drawn. Suprafascial flap harvest, for example, has been associated with lower donor-site morbidity in several comparative studies, probably owing to improved preservation of the paratenon and superficial vascular network [5,6,22]. Nevertheless, despite these encouraging findings, the available evidence remains predominantly retrospective and methodologically heterogeneous, preventing definitive recommendations regarding its routine adoption. Accordingly, the potential advantages of suprafascial dissection should be interpreted cautiously until confirmed by adequately designed prospective comparative studies.

In contrast to tendon exposure, graft loss or failure demonstrated substantial between-study heterogeneity, with a pooled incidence of approximately 11% and a wide prediction interval. This variability most likely reflects differences in donor-site reconstruction techniques, graft type and thickness, postoperative dressing protocols, patient-related risk factors, follow-up duration, and, importantly, the inconsistent definitions used to classify graft-related complications. Whereas some investigators reported partial graft necrosis or incomplete graft take, others evaluated complete graft failure or combined wound-healing complications, substantially limiting direct comparison across studies. Consequently, the pooled incidence should be interpreted as an overall benchmark of reported graft-related morbidity rather than as a universally applicable complication rate.

The present findings do not permit reliable conclusions regarding the comparative effectiveness of split-thickness skin grafts, full-thickness skin grafts, dermal substitutes, or other donor-site reconstruction strategies. Comparative meta-analysis was intentionally avoided because of marked clinical heterogeneity, inconsistent outcome definitions, and the limited number of sufficiently homogeneous studies within each reconstructive category. Importantly, the absence of statistically demonstrable superiority should not be interpreted as evidence of equivalence between techniques. Rather, it reflects the persistent methodological limitations of the available literature. This interpretation is fully consistent with the conclusions reached by the most recent systematic reviews and meta-analyses, all of which emphasize that current evidence is constrained more by study quality and methodological heterogeneity than by the true absence of clinically relevant differences between reconstructive strategies [13,14,15].

From a practical standpoint, each donor-site reconstruction strategy presents specific advantages and limitations. Split-thickness skin grafting (STSG) remains the most widely adopted technique because it is technically straightforward, readily available, and suitable for covering relatively large donor-site defects. Nevertheless, incomplete graft take, contour irregularities, scar contraction, and aesthetic dissatisfaction may occur, particularly in extensive defects [13,14,15,18]. Full-thickness skin grafts (FTSGs) have been proposed to improve scar quality and reduce secondary contraction; however, their applicability may be limited by graft availability, donor-site morbidity, and defect size. Importantly, the three most recent systematic reviews and meta-analyses reached broadly consistent conclusions, indicating that the current evidence remains insufficient to recommend either STSGs or FTSGs as the preferred donor-site closure technique because of the predominance of retrospective studies, heterogeneous surgical protocols, and inconsistent outcome reporting [13,14,15]. In this context, the present findings further support an individualized approach to donor-site reconstruction based on defect characteristics, patient-related factors, and surgeon experience rather than on the presumed superiority of a single reconstructive technique.

Dermal substitutes and collagen-based matrices have emerged as attractive alternatives for donor-site reconstruction because they may improve tissue regeneration, reduce scar contracture, and facilitate wound healing while avoiding some of the limitations associated with conventional skin grafting. However, these techniques often require staged procedures, increase treatment costs, and may prolong the overall healing period [7,11]. Recent comparative evidence has nevertheless provided encouraging results. Galazka et al. demonstrated improved wound-healing characteristics following ulnar-based transposition flap reconstruction compared with conventional STSG closure, suggesting enhanced tissue regeneration and favorable clinical outcomes in selected patients [17]. Although these findings are promising, they derive from a relatively small single-center cohort and therefore require confirmation through larger prospective comparative studies before widespread implementation.

Alternative donor-site closure techniques have also continued to evolve. Primary closure or local flap reconstruction avoids a grafted donor surface and may improve aesthetic appearance and wound healing in appropriately selected patients, although these approaches remain limited by defect dimensions, tissue laxity, and the potential for excessive wound tension [8]. Similarly, recent comparative studies evaluating contemporary closure techniques have emphasized that no single reconstructive strategy consistently outperforms the others across all clinical scenarios. Sabatakakis et al. reported favorable clinical outcomes with two modern donor-site closure techniques but concluded that defect size, local tissue characteristics, and careful patient selection remain the principal determinants of reconstructive success rather than the closure technique itself [16]. These observations further reinforce the concept that donor-site reconstruction should be individualized rather than protocol-driven.

Negative-pressure wound therapy (NPWT) has also been investigated as an adjunctive strategy for donor-site management. Available studies suggest that NPWT may improve graft stabilization, reduce early wound complications, and accelerate initial functional recovery; however, evidence supporting a sustained long-term clinical benefit remains limited [10]. Consequently, although NPWT may represent a useful adjunct in selected high-risk patients, its routine use cannot currently be recommended on the basis of the available evidence alone.

Recent innovations further illustrate the ongoing evolution of donor-site management. Alnemri et al. reported favorable outcomes using a staged reconstructive approach combining a dermal regeneration template with delayed split-thickness skin grafting, achieving lower rates of graft breakdown and tendon exposure compared with immediate skin grafting [18]. Likewise, Zuo et al. described a trilobed RFFF design that enabled primary donor-site closure without skin grafting, with no donor-site complications observed in their case series [12]. Although these reports highlight promising technical innovations, both studies were retrospective and involved carefully selected patient populations. Consequently, their findings should be interpreted cautiously until validated by adequately powered prospective comparative investigations.

Overall, the currently available evidence indicates that donor-site management is undergoing continuous refinement, with increasing emphasis on tissue preservation, biological reconstruction, and patient-centered outcomes. Nevertheless, despite these technical advances, high-quality comparative evidence remains limited, and no contemporary donor-site reconstruction strategy has yet demonstrated clear superiority over established techniques. Accordingly, reconstructive decisions should continue to be guided by defect characteristics, patient-specific factors, available resources, and surgeon expertise rather than by the expectation of universally superior outcomes associated with any single reconstructive approach.

Beyond tendon exposure and graft loss, the present review highlights the broader spectrum of donor-site morbidity that should not be underestimated during reconstructive planning. Delayed wound healing, sensory disturbances, scar-related sequelae, neuropathic pain, and cosmetic dissatisfaction were among the most frequently reported secondary complications. These findings are consistent with previous reports demonstrating that donor-site morbidity extends well beyond wound-related complications and may substantially influence patient perception of surgical success, upper-extremity function, and health-related quality of life [1,24,25,31]. Although these complications rarely compromise flap survival or require major surgical revision, they may delay postoperative recovery, impair return to daily activities, and negatively affect long-term patient satisfaction.

Increasing attention has recently been directed toward patient-reported outcome measures (PROMs), recognizing that successful reconstruction should be evaluated not only according to flap viability but also according to patient-centered outcomes. Functional impairment following RFFF harvest is generally reported to be mild; however, interpretation of the available evidence remains challenging because functional outcomes have been assessed using heterogeneous instruments, including Disabilities of the Arm, Shoulder and Hand (DASH) scores, grip-strength measurements, wrist range of motion, sensory testing, scar assessment scales, and subjective patient-reported symptoms [31]. More recent comparative studies using validated PROMs have likewise demonstrated comparable long-term functional outcomes across different donor-site closure techniques despite differences in specific wound-related complications, further emphasizing the importance of incorporating standardized patient-reported measures into future comparative research [16].

The methodological limitations identified through the JBI critical appraisal should also be considered when interpreting the pooled estimates. The predominance of retrospective observational studies, limited adjustment for confounding factors, heterogeneous patient populations, and inconsistent follow-up protocols may have introduced selection bias and affected complication reporting. Furthermore, donor-site morbidity was frequently evaluated as a secondary outcome rather than a predefined study endpoint, resulting in incomplete reporting of clinically relevant variables such as sensory recovery, scar quality, patient satisfaction, and functional impairment. These limitations reinforce the interpretation of the pooled estimates as benchmark values derived from the currently available literature rather than precise estimates of complication risk applicable to every clinical setting.

The present findings have several clinically relevant implications. First, patients undergoing RFFF reconstruction should be informed that donor-site complications remain relatively common despite the excellent reliability and survival of the flap itself. Based on the pooled estimates, tendon exposure may occur in approximately one of every twelve donor sites, whereas graft loss or failure may affect approximately one of every ten patients. These quantitative estimates may facilitate preoperative counseling and shared decision-making while providing useful benchmark values against which emerging reconstructive strategies can be evaluated. However, because meaningful comparative meta-analysis according to donor-site reconstruction technique was not methodologically feasible, the present results should not be interpreted as supporting one closure technique over another. Rather, the current evidence indicates that donor-site reconstruction should continue to be individualized according to defect characteristics, patient-related factors, reconstructive goals, and surgeon expertise [2,3,4,13,14,15,16,17].

Another important finding emerging from this review is the substantial lack of methodological standardization within the existing literature. Considerable variability exists not only in surgical techniques but also in the definitions of tendon exposure, graft failure, wound-healing complications, follow-up duration, and assessment of functional and aesthetic outcomes. Similar limitations have recently been highlighted by the most comprehensive systematic reviews comparing donor-site reconstruction techniques [13,14,15]. Development of an internationally accepted core outcome set for donor-site assessment following RFFF harvest would substantially improve study comparability, facilitate future evidence synthesis, and enable more robust comparative meta-analyses. Such standardization should include validated functional instruments, objective measurements of upper-extremity performance, scar assessment tools, and patient-reported outcome measures to better capture the overall impact of donor-site morbidity.

The certainty of evidence remained low according to the GRADE framework, reflecting the predominance of retrospective observational studies, inconsistency in outcome reporting, and limited control of confounding variables. Consequently, although the pooled estimates presented in this review appear methodologically robust, they should primarily be regarded as reference benchmarks rather than definitive complication rates. Future research should prioritize adequately powered prospective multicenter comparative studies evaluating donor-site reconstruction techniques using standardized definitions of tendon exposure, graft failure, wound healing, functional recovery, scar quality, aesthetic outcomes, and validated patient-reported outcome measures. Only through methodological standardization and high-quality comparative research will it be possible to determine whether emerging reconstructive strategies—including biological matrices, dermal substitutes, primary closure techniques, tissue-preserving approaches, and negative-pressure wound therapy—provide clinically meaningful advantages over conventional donor-site management [10].

Taken together, the present findings suggest that the principal challenge in contemporary radial forearm free flap reconstruction is no longer flap survival, which has reached consistently excellent outcomes in experienced centers, but rather optimization of donor-site management. Until higher-quality comparative evidence becomes available, reconstructive decisions should remain individualized and based on defect characteristics, patient-specific factors, and surgeon experience rather than on the presumed superiority of any single donor-site closure technique. In this context, the benchmark estimates provided by the present meta-analysis may serve as an important reference for future comparative studies and contribute to the development of more standardized, evidence-based approaches to donor-site reconstruction.

## 5. Limitations

Several limitations of the present review should be acknowledged. First, the available evidence was derived predominantly from retrospective observational studies with relatively small sample sizes, resulting in an overall low certainty of evidence according to the GRADE framework. Consequently, the pooled estimates should be interpreted as benchmark estimates of donor-site morbidity rather than definitive complication rates. Second, substantial clinical and methodological heterogeneity existed across the included studies. Variability in donor-site reconstruction techniques, comparator groups, patient populations, surgical indications, follow-up duration, and outcome definitions precluded meaningful comparative meta-analysis between reconstructive strategies. Accordingly, only pooled incidence analyses were considered methodologically appropriate for quantitative synthesis. Third, donor-site morbidity was frequently reported as a secondary endpoint rather than the primary objective of the original investigations. Consequently, several clinically relevant outcomes—including delayed wound healing, sensory disturbances, scar quality, grip strength, wrist mobility, aesthetic outcomes, and patient-reported quality of life—were assessed inconsistently using heterogeneous measurement instruments, precluding quantitative synthesis and limiting direct comparisons across studies. In addition, the restriction of the literature search to English-language publications may have introduced language bias and may have resulted in the exclusion of potentially relevant studies published in other languages. Fourth, although sensitivity analyses confirmed the robustness of the pooled estimates for the primary outcomes, the limited number of studies within individual reconstructive subgroups prevented meaningful subgroup analyses and meta-regression to explore the potential influence of donor-site reconstruction technique, skin graft type, dressing protocol, postoperative rehabilitation, or patient-related risk factors on donor-site morbidity. Finally, although publication bias was formally assessed when appropriate, evidence of possible small-study effects was observed for tendon exposure. This finding should be interpreted cautiously because conventional methods for detecting publication bias have recognized limitations when applied to single-arm proportion meta-analyses. Despite these limitations, the present review provides one of the most comprehensive quantitative syntheses currently available on donor-site morbidity following radial forearm free flap harvest for head and neck reconstruction. The pooled estimates presented herein may serve as clinically relevant benchmark values for patient counseling, future comparative investigations, and the development of standardized outcome reporting in reconstructive microsurgery.

## 6. Conclusions

Donor-site morbidity remains an important determinant of overall reconstructive success following radial forearm free flap harvest, despite the well-established reliability of the flap in head and neck reconstruction. This systematic review and meta-analysis demonstrates that tendon exposure and graft loss/failure occur in approximately 8% and 11% of donor sites, respectively, providing clinically relevant benchmark estimates for patient counseling, surgical planning, and future comparative research.

Although multiple donor-site reconstruction strategies have been proposed, the available comparative evidence remains insufficient to establish the superiority of any specific technique, and this lack of evidence should not be interpreted as demonstrating equivalence among approaches. Donor-site reconstruction should therefore be individualized according to defect characteristics, patient factors, available tissue, and surgeon experience until higher-quality comparative evidence becomes available. The available literature remains limited by methodological heterogeneity, inconsistent outcome reporting, and the predominance of retrospective observational studies. Future prospective multicenter investigations should adopt standardized definitions of donor-site complications together with validated functional and patient-reported outcome measures to facilitate meaningful comparisons between reconstructive strategies.

Ultimately, improving donor-site outcomes should be considered a fundamental objective of reconstructive surgery rather than a secondary technical consideration. Establishing standardized outcome reporting and generating high-quality comparative evidence will be essential to optimize donor-site management and further refine the role of the radial forearm free flap in contemporary head and neck reconstruction.

## Figures and Tables

**Table 1:** Features of the 22 included studies. NR, not reported; NA, not applicable.

Author, Year, Country, Study Design	Sample Size	Follow-up	Tendon Exposure (n/N); Graft Loss/Failure (n/N)	Other Donor-Site Complications
Ki et al. (2023), South Korea, Retrospective comparative study [5]	32 patients/donor sites	Mean 8 months	3/32; 6/32	Delayed healing 13/32; secondary skin graft 1/32
Shonka et al. (2017), USA, Retrospective comparative study [6]	47 patients/donor sites	Donor site evaluated at 6 days, 2 weeks, and 1 month	5/47; NR	Donor-site hematoma 3/47
Alnemri et al. (2026), USA, Retrospective comparative study [18]	179 patients/donor sites	Variable; cohort May 2012–April 2023	14/179; 18/179	Hematoma 2; seroma 8; infection/cellulitis 9; hypertrophic scarring 8; thenar paresthesia 7; extremity weakness 5; excessive scar contracture 2
Schwarzer et al. (2016), Germany, Prospective randomized clinical trial [21]	50 randomized; 41 evaluable at endpoint	3 months	NR; 1/41	Dysesthesia, pain, wound complications, impaired mobility, impaired strength, graft shrinkage
Garg et al. (2017), USA, Retrospective comparative study [22]	75 patients/donor sites	NR	8/75; NR *	Delayed healing 19/75; infection 2/75. * Delayed healing included epidermolysis, wound breakdown, graft separation or graft failure, but graft failure was not separately extractable.
Cherubino et al. (2020), Italy, Retrospective comparative study [7]	21 patients/donor sites	1 year	1/21; 4/21	Wound infection 4/21; hand dysesthesia 1/21; DASH functional assessment reported
Potet et al. (2020), France, Retrospective comparative study [8]	101 patients/donor sites	Minimum 12 months	10/101; 16/65	Infection 6; hematoma 5; wound reopening 6; edema/epidermolysis 4; sensory disturbances reported
Zuidam et al. (2005), Netherlands, Retrospective comparative study [23]	34 patients/donor sites	Mean 25–26 months	NR; 1/34	Superficial/partial necrosis 1/34; hypergranulation 2; hypertrophic scar 2; epidermolysis 2; DASH and functional assessment reported
Van Kouwenberg et al. (2019), USA, Retrospective comparative cohort study [24]	40 patients/donor sites	Mean 119 days (ERFFF) and 254 days (RFFF)	1/40; 2/40	Numbness 12/40; delayed wound healing 2/40; hypertrophic scar 1/40; CRPS 1/40
Satteson et al. (2017), USA, Retrospective comparative study [25]	77 patients/donor sites (25 ORFFF, 52 FRFFF)	Mean 14.2 months (ORFFF) and 11.7 months (FRFFF)	10/77; 5/77	Radius fracture 1; hardware infection 1; no soft tissue infection or sensory neuropathy reported
Park et al. (2015), South Korea, Retrospective case series [9]	22 patients/donor sites	Mean 34.9 months	1/22; NA	No graft rejection/failure, infection, hand dysfunction or hand swelling; complete healing in 21/22 by 1.5–3 months
Hong (2024), South Korea, Retrospective cohort study [26]	67 patients/donor sites	Median 8 months; minimum 6 months	6/67; NR *	Delayed wound healing 25/67; seroma 4/67; hematoma 10/67; infection 3/67; wound dehiscence 1/67; paresthesia 9/67; wrist stiffness 3/67; hand swelling 3/67; reduced strength 2/67. * Partial graft loss not separately extractable.
Chen et al. (2005), Taiwan, Retrospective case series [27]	38 donor sites in 37 patients	Mean 14 months; range 5–26 months	2/38; 4/38	Abnormal sensation 10/38; poor appearance 3/38; reduced grip strength 4/38
Wirthmann et al. (2014), Switzerland, Prospective follow-up study [28]	13 patients/donor sites	Mean 23.8 months; range 6–51 months	0/13; 1/13	Unstable scar 2; cold intolerance 2; reduced/partially intact sensibility 7; scar contracture cords 2; delayed return to work 4
Orlik et al. (2014), Canada, Cross-sectional long-term follow-up study [29]	9 long-term survivors	Mean 50 months; range 26–109 months	NR; NR	Forearm fracture 1; stitch abscess 1; cold intolerance 4; reduced sensation, pronation and manual dexterity reported
de Bree et al. (2004), Netherlands, Cross-sectional follow-up study [30]	37 patients/donor sites	Long-term follow-up; exact duration NR	NR; NR	Impaired function 9/37; inability to wear watch/bracelet 14/37; numbness 17/37; soreness 5/37; itching 5/37; cold intolerance 6/37; poor appearance 5/37
Lee & Lee (2023), South Korea, Retrospective case series [31]	5 patients/donor sites	Mean 15.7 months; range 9–37 months	0/5; NA	No delayed wound healing, dehiscence, infection, hematoma, seroma, flap loss, contracture, adhesion, scar revision or debulking
Hanna et al. (2014), USA, Retrospective case series [32]	50 patients/donor sites	Until complete healing; long-term clinical photographs reported	NR; 15/50	Less than 100% graft take in 15/50; minor office debridement 1/50; no surgical revision of forearm donor site; no recipient-site infections
Zuo et al. (2025), China, Retrospective longitudinal case series [12]	16 patients/donor sites	Mean 38.6 months; range 12–40 months	0/16; NA	No donor-site complications; no flap necrosis; no skin graft required; DASH and UW-QoL reported
Halama et al. (2019), Germany, Prospective randomized controlled trial [10]	50 patients/donor sites (VAC n = 25, conventional dressing n = 25)	8 weeks	NR; NR	Faster grip-strength recovery with VAC; wound-area reduction and wrist movement measured, but complication event rates not reported
Pirlich et al. (2018), Germany, Retrospective comparative study [33]	39 patients/donor sites (indirect closure n = 18, direct closure n = 21)	Long-term follow-up; exact duration NR	NR; NR *	Wound-healing disorders: 2/18 vs 5/21; surgical revision 1/18 vs 1/21. * Type of wound-healing disorder not sufficiently specified for graft-failure extraction.
Fang et al. (2014), China, Prospective matched cohort study [34]	52 RFFF patients/donor sites	>=1 year	NR; 4/52	Partial skin graft necrosis 4/52; no total graft failures; hematoma requiring immediate re-exploration 3; postoperative DASH score worsening

* Indicates that skin graft loss/failure could not be extracted as a separate outcome according to the predefined definitions adopted in this systematic review. See the corresponding notes for study-specific details.

## Data Availability

The data that support the findings of this study are available from the corresponding author upon reasonable request.

## Supplementary Materials

The following supporting information can be downloaded at: https://www.mdpi.com/article/10.3390/jcm15176513/s1, Supplementary S1 (complete search strategies), Supplementary Figures S1–S7 (additional meta-analytic and risk of bias analyses), Supplementary Table S1 (PRISMA 2020 checklist), Supplementary Table S2 (GRADE Summary of Findings) and Supplementary Table S3 (detailed characteristics of included studies, donor-site reconstruction techniques, and outcome-specific data) are available online.

## Abbreviations

The following abbreviations are used in this manuscript:

CI	Confidence interval
DASH	Disabilities of the Arm, Shoulder and Hand
GRADE	Grading of Recommendations Assessment, Development and Evaluation
GLMM	Generalized linear mixed model
JBI	Joanna Briggs Institute
ML	Maximum likelihood
NR	Not reported
NA	Not applicable
PRISMA	Preferred Reporting Items for Systematic Reviews and Meta-Analyses
PROSPERO	International Prospective Register of Systematic Reviews
QoL	Quality of life
ROM	Range of motion
RCT	Randomized controlled trial
RFFF	Radial forearm free flap
RR	Risk ratio
STSG	Split-thickness skin graft
FTSG	Full-thickness skin graft
τ^2^	Between-study variance

## References

[1] Brown M.T., Couch M.E., Huchton D.M. (1999). Assessment of donor-site functional morbidity from radial forearm fasciocutaneous free flap harvest. Arch. Otolaryngol. Head. Neck Surg..

[2] Loeffelbein D.J., Al-Benna S., Steinsträßer L., Satanovskij R.M., Rohleder N.H., Mücke T., Wolff K.D., Kesting M.R. (2012). Reduction of donor site morbidity of free radial forearm flaps: What level of evidence is available?. Eplasty.

[3] Patel R. (2022). Reducing morbidity in radial forearm free flap donor site: A review of closure techniques. Curr. Opin. Otolaryngol. Head. Neck Surg..

[4] Fatani B. (2023). Radial forearm free flap for head and neck defect reconstruction: An up-to-date review of the literature. Cureus.

[5] Ki S.H., Park T.J., Yoon J.M. (2023). Surgical outcomes of suprafascial and subfascial radial forearm free flaps in head and neck reconstruction. Arch. Craniofac Surg..

[6] Shonka D.C., Kohli N.V., Milam B.M., Jameson M.J. (2017). Suprafascial harvest of the radial forearm free flap decreases the risk of postoperative tendon exposure. Ann. Otol. Rhinol. Laryngol..

[7] Cherubino M., Magni A., Turri-Zanoni M., Garutti L., Di Summa P., Campisi C., Valdatta L. (2020). Radial forearm free flap in head and neck cancer treatment: May dermal substitutes have a role in minimizing donor-site morbidity?. Eur. J. Plast. Surg..

[8] Potet P., De Bonnecaze G., Chabrillac E., Dupret-Bories A., Vergez S., Chaput B. (2020). Closure of radial forearm free flap donor site: A comparative study between keystone flap and skin graft. Head. Neck..

[9] Park T.J., Kim H.J., Ahn K.M. (2015). Double-layered collagen graft to the radial forearm free flap donor site without skin graft. Maxillofac. Plast. Reconstr. Surg..

[10] Halama D., Dreilich R., Lethaus B., Bartella A., Pausch N.C. (2019). Donor-site morbidity after harvesting of radial forearm free flaps: Comparison of vacuum-assisted closure with conventional wound care: A randomized controlled trial. J. Cranio-Maxillofac. Surg..

[11] Shah R., Rodrigues R., Phillips V., Khatib M. (2024). The use of artificial dermal substitutes for repair of the donor site following harvesting of a radial forearm free flap: A systematic review. J. Plast. Reconstr. Aesthet. Surg..

[12] Zuo L., Jiang Z., Liu J., Tian H., Gao S., Huang W. (2025). A trilobed radial forearm free flap: A novel approach to oral cavity reconstruction. Head Neck.

[13] Zhang C., Pandya S., Alessandri Bonetti M., Costantino A., Egro F.M. (2024). Comparison of split-thickness skin graft versus full-thickness skin graft for radial forearm flap donor site closure: A systematic review and meta-analysis. Am. J. Otolaryngol..

[14] Moors J.J.E., Xu Z., Xie K., Rashad A., Vladu O., Egger J., Röhrig R., Hölzle F., Puladi B. (2025). Full-thickness skin graft versus split-thickness skin graft for fasciocutaneous radial forearm free flap donor site closure: A systematic review and meta-analysis. Syst. Rev..

[15] Saleki M., Noor M.A., Hurt P., Abul A. (2023). Full-thickness skin graft versus split-thickness skin graft for radial forearm free flap transfer in oral cavity reconstruction: A systematic review and meta-analysis. Cureus.

[16] Sabatakakis A., Fenske J., Lampert P., Kreiker H., Steffen C., Koerdt S., Doll C., Kreutzer K., Heiland M., Rendenbach C. (2025). Towards optimized donor site closure of radial forearm flaps: A cohort study of two techniques. J. Cranio-Maxillofac. Surg..

[17] Galazka A., Stawarz K., Bienkowska-Pluta K., Paszkowska M., Misiak-Galazka M. (2025). Optimizing wound healing in radial forearm donor sites: A comparative study of ulnar-based flap and split-thickness skin grafting. Biomedicines.

[18] Alnemri A., Moroco A., Garg N., Bridgham K., Davis M., Kaki P., McCann A., Thal A., Krein H., Heffelfinger R. (2026). Timing of split-thickness skin grafting for radial forearm free flaps on donor-site morbidity. Microsurgery.

[19] Page M.J., McKenzie J.E., Bossuyt P.M., Boutron I., Hoffmann T.C., Mulrow C.D., Shamseer L., Tetzlaff J.M., Akl E.A., Brennan S.E. (2021). The PRISMA 2020 statement: An updated guideline for reporting systematic reviews. BMJ.

[20] Munn Z., Barker T.H., Moola S., Tufanaru C., Stern C., McArthur A., Stephenson M., Aromataris E. (2020). Methodological quality of case series studies: An introduction to the JBI critical appraisal tool. JBI Evid. Synth..

[21] Schwarzer C., Mücke T., Wolff K.D., Loeffelbein D.J., Rau A. (2016). Donor site morbidity and flap perfusion of subfascial and suprafascial radial forearm flaps: A randomized prospective clinical comparison trial. J. Cranio-Maxillofac. Surg..

[22] Garg R.K., Wieland A.M., Poore S.O., Sanchez R., Hartig G.K. (2017). The radial forearm snake flap: A novel approach to oral cavity and oropharyngeal reconstruction that reduces forearm donor site morbidity. Microsurgery.

[23] Zuidam J.M., Coert J.H., Hofer S.O. (2005). Closure of the donor site of the free radial forearm flap: A comparison of full-thickness graft and split-thickness skin graft. Ann. Plast. Surg..

[24] Van Kouwenberg E.A., Yan A., Patel A., McLaughlin R.L., Northrup P., Cintron M., Agag R.L. (2019). Endoscopic-assisted radial forearm free flap harvest: A novel technique to reduce donor-site morbidity. J. Reconstr. Microsurg..

[25] Satteson E.S., Satteson A.C., Waltonen J.D., Li Z., Wiesler E.R., Apel P.J., Graves B.R. (2017). Donor-site outcomes for the osteocutaneous radial forearm free flap. J. Reconstr. Microsurg..

[26] Hong S. (2024). Risk factors for postoperative donor site complications in radial forearm free flaps. Medicina.

[27] Chen C.M., Lin G.T., Fu Y.C., Shieh T.Y., Huang I.Y., Shen Y.S., Chen C.H. (2005). Complications of free radial forearm flap transfers for head and neck reconstruction. Oral. Surg. Oral. Med. Oral. Pathol. Oral. Radiol. Endod..

[28] Wirthmann A., Finke J.C., Giovanoli P., Lindenblatt N. (2014). Long-term follow-up of donor site morbidity after defect coverage with Integra following radial forearm flap elevation. Eur. J. Plast. Surg..

[29] Orlik J.R., Horwich P., Bartlett C., Trites J., Hart R., Taylor S.M. (2014). Long-term functional donor site morbidity of the free radial forearm flap in head and neck cancer survivors. J. Otolaryngol. Head. Neck Surg..

[30] de Bree R., Hartley C., Smeele L.E., Kuik D.J., Quak J.J., Leemans C.R. (2004). Evaluation of donor site function and morbidity of the fasciocutaneous radial forearm flap. Laryngoscope.

[31] Lee J.K., Lee K.T. (2023). Coverage of radial forearm free flap donor site defect using another free flap. Microsurgery.

[32] Hanna T.C., McKenzie W.S., Holmes J.D. (2014). Full-thickness skin graft from the neck for coverage of the radial forearm free flap donor site. J. Oral. Maxillofac. Surg..

[33] Pirlich M., Horn I.S., Mozet C., Pirlich M., Dietz A., Fischer M. (2018). Functional and cosmetic donor site morbidity of the radial forearm free flap: Comparison of two different coverage techniques. Eur. Arch. Otorhinolaryngol..

[34] Fang Q.G., Shi S., Zhang X., Li Z.N., Liu F.Y., Sun C.F. (2014). Upper extremity morbidity after radial forearm flap harvest: A prospective study. J. Int. Med. Res..

[35] Xu Q., Chen P.L., Liu Y.H., Wang S.M., Xu Z.F., Feng C.J. (2022). Comparing donor site morbidity between radial and ulnar forearm free flaps: A meta-analysis. Br. J. Oral Maxillofac. Surg..

[36] Ranganath K., Jalisi S.M., Naples J.G., Gomez E.D. (2022). Comparing outcomes of radial forearm free flaps and anterolateral thigh free flaps in oral cavity reconstruction: A systematic review and meta-analysis. Oral Oncol..

