# Supplementary material for: Donor-Site Morbidity Following Radial Forearm Free Flap Harvest for Head and Neck Reconstruction: A Systematic Review and Meta-Analysis
Source: J Clin Med. 2026 Aug 23;15(17):6513. doi: 10.3390/jcm15176513 (PMC13565850; doi:10.3390/jcm15176513)
Supplement: Supplementary file 1 [file jcm-15-06513-s001.zip › jcm-4459374-Supplementary Figures S1–S7 caption.pdf]

Supplementary Figures S1–S7 (additional meta-analytic and risk of bias analyses)
