# Supplementary material for: Donor-Site Morbidity Following Radial Forearm Free Flap Harvest for Head and Neck Reconstruction: A Systematic Review and Meta-Analysis
Source: J Clin Med. 2026 Aug 23;15(17):6513. doi: 10.3390/jcm15176513 (PMC13565850; doi:10.3390/jcm15176513)
Supplement: Supplementary file 1 [file jcm-15-06513-s001.zip › Supplementary S1 (complete search strategies) copia.pdf]

PubMed/MEDLINE

```
(
  "radial forearm free flap"[Title/Abstract]
  OR "radial forearm flap"[Title/Abstract]
  OR RFFF[Title/Abstract]
)
AND
(
  "head and neck"[Title/Abstract]
  OR "oral cavity"[Title/Abstract]
  OR "head and neck neoplasms"[MeSH]
)
AND
(
  "donor site"[Title/Abstract]
  OR "donor-site"[Title/Abstract]
  OR "donor site morbidity"[Title/Abstract]
  OR "donor site complication*"[Title/Abstract]
  OR "tendon exposure"[Title/Abstract]
  OR "graft loss"[Title/Abstract]
  OR "wound healing"[Title/Abstract]
  OR "functional outcome*"[Title/Abstract]
  OR "wrist function"[Title/Abstract]
  OR "grip strength"[Title/Abstract]
  OR DASH[Title/Abstract]
  OR aesthetic[Title/Abstract]
  OR scar[Title/Abstract]
)
AND ("2000/01/01"[Date - Publication] : "2026/06/14"[Date - Publication])
```

Search period: January 2000 to June 2026.

Embase

(

'radial forearm free flap':ti,ab,kw

OR 'radial forearm flap':ti,ab,kw

OR rfff:ti,ab,kw

)

AND

(

'head and neck reconstruction':ti,ab,kw

OR 'head and neck cancer':ti,ab,kw

OR 'oral cavity':ti,ab,kw

OR 'head and neck tumor'/exp

)

AND

(

'donor site':ti,ab,kw

OR 'donor site morbidity':ti,ab,kw

OR 'donor site complication':ti,ab,kw

OR 'tendon exposure':ti,ab,kw

OR 'graft loss':ti,ab,kw

OR 'wound healing':ti,ab,kw

OR 'functional outcome':ti,ab,kw

OR 'wrist function':ti,ab,kw

OR 'grip strength':ti,ab,kw

OR DASH:ti,ab,kw

OR aesthetic:ti,ab,kw

OR scar:ti,ab,kw

)

AND [2000-2026]/py

AND [english]/lim

AND [article]/lim

Search period: January 2000 to June 2026.

Scopus

TITLE-ABS-KEY("radial forearm free flap" OR "radial forearm flap" OR RFFF)

AND TITLE-ABS-KEY("head and neck reconstruction" OR "oral cavity reconstruction" OR "oral cancer")

AND TITLE-ABS-KEY(complication* OR morbidity OR "flap failure" OR thrombosis OR fistula OR infection OR dehiscence OR "donor site morbidity")

AND PUBYEAR > 1999

Search period: January 2000 to June 2026.
