# Supplementary material for: Donor-Site Morbidity Following Radial Forearm Free Flap Harvest for Head and Neck Reconstruction: A Systematic Review and Meta-Analysis
Source: J Clin Med. 2026 Aug 23;15(17):6513. doi: 10.3390/jcm15176513 (PMC13565850; doi:10.3390/jcm15176513)
Supplement: Supplementary file 1 [file jcm-15-06513-s001.zip › Supplementary Table S2 copia.pdf]

Supplementary Table S2

Summary of Findings and GRADE certainty of evidence assessment for the primary outcomes following radial forearm free flap donor-site reconstruction.

Outcome	Studies	Donor sites	Pooled incidence (95% CI)	I^2	Risk of bias	Inconsistency	Indirectness	Imprecision	Publication bias	Overall certainty
Tendon exposure	14	733	8% (95% CI, 6–11%)	0%	Serious[1]	Not serious[2]	Not serious	Not serious	Not downgraded[3]	⊕⊕◯◯ Low
Graft loss/failure	12	642	11% (95% CI, 7–16%)	67.1%	Serious[1]	Serious[4]	Not serious	Not serious	Not downgraded[3]	⊕⊕◯◯ Low

Footnotes

[1] The body of evidence was predominantly derived from observational studies and was affected by methodological limitations identified during the JBI risk-of-bias assessment, including incomplete follow-up reporting and limited control of potential confounding.

[2] Although the point estimate of heterogeneity was $I^2 = 0\%$, its 95% CI ranged from 0% to 55%. No additional downgrade for inconsistency was applied because the prediction interval was relatively narrow (6.37–10.81%) and leave-one-out sensitivity analysis demonstrated stability of the pooled estimate.

[3] No additional downgrade for publication bias was applied. Although funnel-plot asymmetry and Egger's regression test suggested possible small-study effects, these methods have limited interpretability in single-arm proportional meta-analyses, in which a conventional distinction between "positive" and "negative" studies is not clearly defined.

[4] Inconsistency was considered serious for graft loss/failure because substantial between-study heterogeneity was observed ($I^2 = 67.1\%$, 95% CI 39.6–82.0%), together with a wide 95% prediction interval (2.40–36.15%). Although leave-one-out sensitivity analysis demonstrated stability of the pooled point estimate, substantial variability in the underlying incidence across studies remained.
