# Supplementary material for: Donor-Site Morbidity Following Radial Forearm Free Flap Harvest for Head and Neck Reconstruction: A Systematic Review and Meta-Analysis
Source: J Clin Med. 2026 Aug 23;15(17):6513. doi: 10.3390/jcm15176513 (PMC13565850; doi:10.3390/jcm15176513)
Supplement: Supplementary file 1 [file jcm-15-06513-s001.zip › Supplementary Table S3.pdf]

Table S3. Characteristics of included studies and donor-site outcomes following radial forearm free flap harvest

Event definitions: tendon exposure was extracted only when a numerical event count or explicit zero events was reported. Graft loss/failure included partial or total skin graft loss, necrosis, incomplete graft take, or graft breakdown when numerically reported. NA indicates no skin graft was used; NR indicates not reported or not extractable.

Author (Year)	Country	Study design	Sample size	Indication	Donor-site coverage technique	Follow-up	Tendon exposure (n/N)	Graft loss/failure (n/N)	Other donor-site complications	Tendon MA	Graft MA
Ki et al. (2023)	South Korea	Retrospective comparative study	32 patients/donor sites	Head and neck reconstruction (mostly SCC)	STSG in all patients; ADM used in some cases; subfascial vs suprafascial harvest	Mean 8 months	3/32	6/32	Delayed healing 13/32; secondary skin graft 1/32	Yes	Yes
Shonka et al. (2017)	USA	Retrospective comparative study	47 patients/donor sites	Head and neck defects	STSG + bolster dressing; subfascial vs suprafascial harvest	Donor site evaluated at 6 days, 2 weeks and 1 month	5/47	NR	Donor-site hematoma 3/47	Yes	No
Alnemri et al. (2026)	USA	Retrospective comparative study	179 patients/donor sites	Head and neck reconstruction	Primary STSG vs staged Integra + STSG	Variable; cohort May 2012-April 2023	14/179	18/179	Hematoma 2; seroma 8; infection/cellulitis 9; hypertrophic scarring 8; thenar paresthesia 7; extremity weakness 5; excessive scar contracture 2	Yes	Yes
Schwarzer et al. (2016)	Germany	Prospective randomized clinical trial	50 randomized; 41 evaluable at endpoint	Oral cavity SCC requiring RFFF reconstruction	FTSG from groin; subfascial vs suprafascial harvest	3 months	NR	1/41	Dysesthesia, pain, wound complications, impaired mobility, impaired strength, graft shrinkage	No	Yes
Garg et al. (2015)	USA	Retrospective comparative study	75 patients/donor sites	Oral cavity and oropharyngeal reconstruction	Snake flap with primary closure (n=18) vs STSG closure (n=57)	NR	8/75	NR*	Delayed healing 19/75; infection 2/75. *Delayed healing included epidermolysis, wound breakdown, graft separation or graft failure, but graft failure was not separately extractable.	Yes	No
Cherubino et al. (2019)	Italy	Retrospective comparative study	21 patients/donor sites	Head and neck cancer reconstruction	Integra + delayed STSG (n=10) vs FTSG (n=11)	1 year	1/21	4/21	Wound infection 4/21; hand dysesthesia 1/21; DASH functional assessment reported	Yes	Yes
Potet et al. (2019)	France	Retrospective comparative study	101 patients/donor sites	Head and neck tumor reconstruction	Keystone flap (n=36) vs skin graft (n=65)	Minimum 12 months	10/101	16/65	Infection 6; hematoma 5; wound reopening 6; edema/epidermolysis 4; sensory disturbances reported	Yes	Yes
Zuidam et al. (2005)	Netherlands	Retrospective comparative study	34 patients/donor sites	Head and neck cancer reconstruction	STSG (n=15) vs ipsilateral forearm FTSG with V-Y closure (n=19)	Mean 25-26 months	NR	1/34	Superficial/partial necrosis 1/34; hypergranulation 2; hypertrophic scar 2; epidermolysis 2; DASH and functional assessment reported	No	Yes
Van Kouwenberg et al. (2018)	USA	Retrospective comparative cohort study	40 patients/donor sites	Head and neck reconstruction (39 intraoral defects, 1 periorbital wound)	Endoscopic-assisted RFFF vs conventional RFFF; mostly Integra + delayed STSG	Mean 119 days (ERFFF) and 254 days (RFFF)	1/40	2/40	Numbness 12/40; delayed wound healing 2/40; hypertrophic scar 1/40; CRPS 1/40	Yes	Yes
Satteson et al. (2017)	USA	Retrospective comparative study	77 patients/donor sites (25 ORFFF, 52 FRFFF)	Head and neck reconstruction	STSG + negative-pressure dressing; ORFFF with prophylactic radial plating	Mean 14.2 months (ORFFF) and 11.7 months (FRFFF)	10/77	5/77	Radius fracture 1; hardware infection 1; no soft tissue infection or sensory neuropathy reported	Yes	Yes
Park et al. (2015)	South Korea	Retrospective case series	22 patients/donor sites	Oral cancer reconstruction (21 SCC, 1 melanoma)	Double-layered collagen graft (MatriDerm + Terudermis), no skin graft; secondary intention healing	Mean 34.9 months	1/22	NA	No graft rejection/failure, infection, hand dysfunction or hand swelling; complete healing in 21/22 by 1.5-3 months	Yes	No
Hong (2024)	South Korea	Retrospective cohort study	67 patients/donor sites	Head and neck cancer reconstruction	STSG with or without ADM (CGDerm, MegaDerm, MatriDerm) + NPWT	Median 8 months; minimum 6 months	6/67	NR*	Delayed wound healing 25/67; seroma 4/67; hematoma 10/67; infection 3/67; wound dehiscence 1/67; paresthesia 9/67; wrist stiffness 3/67; hand swelling 3/67; reduced strength 2/67. *Partial graft loss not separately extractable.	Yes	No
Chen et al. (2005)	Taiwan	Retrospective case series	38 donor sites in 37 patients	Head and neck cancer reconstruction	STSG from thigh	Mean 14 months; range 5-26 months	2/38	4/38	Abnormal sensation 10/38; poor appearance 3/38; reduced grip strength 4/38	Yes	Yes
Wirthmann et al. (2014)	Switzerland	Prospective follow-up study	13 patients/donor sites	Head and neck reconstruction (n=9), phalloplasty (n=3), lower extremity defect (n=1)	Integra + delayed STSG	Mean 23.8 months; range 6-51 months	0/13	1/13	Unstable scar 2; cold intolerance 2; reduced/partially intact sensibility 7; scar contracture cords 2; delayed return to work 4	Yes	Yes
Orlik et al. (2014)	Canada	Cross-sectional long-term follow-up study	9 long-term survivors	Head and neck cancer reconstruction	STSG from thigh	Mean 50 months; range 26-109 months	NR	NR	Forearm fracture 1; stitch abscess 1; cold intolerance 4; reduced sensation, pronation and manual dexterity reported	No	No
de Bree et al. (2004)	Netherlands	Cross-sectional follow-up study	37 patients/donor sites	Head and neck cancer reconstruction	STSG from upper thigh	Long-term follow-up; exact duration NR	NR	NR	Impaired function 9/37; inability to wear watch/bracelet 14/37; numbness 17/37; soreness 5/37; itching 5/37; cold intolerance 6/37; poor appearance 5/37	No	No
Lee & Lee (2023)	South Korea	Retrospective case series	5 patients/donor sites	Head and neck SCC reconstruction (n=4) and penile reconstruction (n=1)	Domino free flap coverage of RFFF donor site (ALT flap n=4, SCIP flap n=1)	Mean 15.7 months; range 9-37 months	0/5	NA	No delayed wound healing, dehiscence, infection, hematoma, seroma, flap loss, contracture, adhesion, scar revision or debulking	Yes	No
Hanna et al. (2014)	USA	Retrospective case series	50 patients/donor sites	Oral, head and neck cancer reconstruction	FTSG harvested from the neck dissection incision	Until complete healing; long-term clinical photographs reported	NR	15/50	Less than 100% graft take in 15/50; minor office debridement 1/50; no surgical revision of forearm donor site; no recipient-site infections	No	Yes
Zuo et al. (2025)	China	Retrospective longitudinal case series	16 patients/donor sites	T2 oral cavity SCC reconstruction	Trilobed RFFF with primary donor-site closure; no skin graft	Mean 38.6 months; range 12-40 months	0/16	NA	No donor-site complications; no flap necrosis; no skin graft required; DASH and UW-QoL reported	Yes	No
Halama et al. (2019)	Germany	Prospective randomized controlled trial	50 patients/donor sites (VAC n=25, conventional dressing n=25)	Orofacial/head and neck cancer reconstruction	FTSG from groin + VAC vs conventional bolster dressing	8 weeks	NR	NR	Faster grip-strength recovery with VAC; wound-area reduction and wrist movement measured, but complication event rates not reported	No	No
Pirlich et al. (2018)	Germany	Retrospective comparative study	39 patients/donor sites (indirect closure n=18, direct closure n=21)	Head and neck reconstruction	Indirect/distant FTSG from groin vs direct/local FTSG/direct closure technique	Long-term follow-up; exact duration NR	NR	NR*	Wound-healing disorders: 2/18 vs 5/21; surgical revision 1/18 vs 1/21. *Type of wound-healing disorder not sufficiently specified for graft-failure extraction.	No	No
Fang et al. (2014)	China	Prospective matched cohort study	52 RFFF patients/donor sites	Head and neck cancer reconstruction	FTSG from abdomen	>=1 year	NR	4/52	Partial skin graft necrosis 4/52; no total graft failures; hematoma requiring immediate re-exploration 3; postoperative DASH score worsening	No	Yes

Summary of quantitative inclusion: 22 studies were included in the systematic review; 14 studies were eligible for tendon exposure meta-analysis and 12 studies were eligible for graft loss/failure meta-analysis. Garg et al. (2015), Hong (2024), and Pirlich et al. (2018) were not included in the graft loss/failure meta-analysis because graft-related outcomes were not separately or sufficiently defined.

Abbreviations: ADM, acellular dermal matrix; ALT, anterolateral thigh; CRPS, complex regional pain syndrome; DASH, Disabilities of the Arm, Shoulder and Hand; ERFFF, endoscopic-assisted radial forearm free flap; FRFFF, fasciocutaneous radial forearm free flap; FTSG, full-thickness skin graft; MA, meta-analysis; NA, not applicable; NPWT, negative-pressure wound therapy; NR, not reported/not extractable; ORFFF, osteocutaneous radial forearm free flap; RFFF, radial forearm free flap; SCIP, superficial circumflex iliac artery perforator; SCC, squamous cell carcinoma; STSG, split-thickness skin graft; VAC, vacuum-assisted closure.
